# A case-control study of exposure to organophosphate flame retardants and risk of thyroid cancer in women

**DOI:** 10.1186/s12885-018-4553-9

**Published:** 2018-06-05

**Authors:** Nicole C. Deziel, Huangdi Yi, Heather M. Stapleton, Huang Huang, Nan Zhao, Yawei Zhang

**Affiliations:** 1 Department of Environmental Health Sciences, Yale School of Public Health, 60 College St, New Haven, CT 06520 USA; 2 Nicholas School of the Environment, Duke University, 9 Circuit Dr, Durham, NC 27710 USA; 3 Department of Surgery, Yale School of Medicine, 333 Cedar St, New Haven, CT 06510 USA; 4 Department of Biostatistics, Yale School of Public Health, 60 College St, New Haven, CT 06520 USA

**Keywords:** Thyroid cancer, Flame retardants, Endocrine disruptor, Women’s health, Environmental exposures, Biomarkers

## Abstract

**Background:**

Growing evidence demonstrates that exposure to organophosphate flame retardants (PFRs) is widespread and that these chemicals can alter thyroid hormone regulation and function. We investigated the relationship between PFR exposure and thyroid cancer and whether individual or temporal factors predict PFR exposure.

**Methods:**

We analyzed interview data and spot urine samples collected in 2010–2013 from 100 incident female, papillary thyroid cancer cases and 100 female controls of a Connecticut-based thyroid cancer case-control study. We measured urinary concentrations of six PFR metabolites with mass spectrometry. We estimated odds ratios (OR) and 95% confidence intervals (95% CI) for continuous and categories (low, medium, high) of concentrations of individual and summed metabolites, adjusting for potential confounders. We examined relationships between concentrations of PFR metabolites and individual characteristics (age, smoking status, alcohol consumption, body mass index [BMI], income, education) and temporal factors (season, year) using multiple linear regression analysis.

**Results:**

No PFRs were significantly associated with papillary thyroid cancer risk. Results remained null when stratified by microcarcinomas (tumor diameter ≤ 1 cm) and larger tumor sizes (> 1 cm). We observed higher urinary PFR concentrations with increasing BMI and in the summer season.

**Conclusions:**

Urinary PFR concentrations, measured at time of diagnosis, are not linked to increased risk of thyroid cancer. Investigations in a larger population or with repeated pre-diagnosis urinary biomarker measurements would provide additional insights into the relationship between PFR exposure and thyroid cancer risk.

**Electronic supplementary material:**

The online version of this article (10.1186/s12885-018-4553-9) contains supplementary material, which is available to authorized users.

## Background

Thyroid cancer is the most common endocrine malignancy worldwide and is three times more common in women compared to men [1, 2]. The age-adjusted annual incidence rate for thyroid cancer in women in the United States (U.S.) nearly tripled over the previous twenty years, from 7.7/10^5^ in 1991 to 22.2/10^5^ in 2014 in women [1]. Papillary thyroid carcinoma accounts for approximately 90% of all thyroid cancer cases in the United States [1].

Though the prognosis for thyroid cancer is quite good (> 90% survival after 20 years) [1], the costs for diagnosis, treatment, and continued surveillance are significant, estimated at $1.6 billion for all U.S. patients diagnosed after 1985 [3]. With the incidence rate climbing rapidly, projected societal costs for 2030 exceed $3.5 billion. Furthermore, thyroid cancer is associated with an increased risk of developing a second primary cancer, potentially attributable to radiation-based treatments such as radioiodine or directed beam radiation therapy [4, 5]. The quality of life of thyroid cancer survivors may be impaired due to co-morbidities and dependence on long-term treatment [6].

The etiology of thyroid cancer is largely unknown. The major established causal factor is exposure to ionizing radiation during childhood [7, 8]; other risk factors include family history of thyroid cancer, personal history of benign thyroid diseases [9], and greater body weight and height [10, 11]. These known and suspected risk factors explain only a small portion of thyroid cancer cases. Changes in diagnostic procedures and increased medical attention to thyroid nodules explain part of the rise during the past two decades [12–14]. However, reported incidence rates have increased across all tumor sizes [15, 16], in younger age groups that are less likely to receive imaging exams [17, 18], and for follicular thyroid cancer, which is less easily detected by newer diagnostic technologies [19]. Taken together, this supports the role of other factors in addition to increased detection [20]. Environmental and occupational exposures to chemicals have been proposed as potentially important contributors to the increasing trend [21–24].

Organophosphate flame retardants have been commonly used in the 1990s and 2000s, and their use has been increasing due to the phase out of the polybrominated diphenyl ether (PBDE) flame retardants [25–27]. Firemaster 550®, a mixture of phosphorous-containing compounds including triphenyl phosphate (TPHP), has gained popularity as an additive to polyurethane foam [26, 28, 29]. Tris (1,3-dichloro-isopropyl) phosphate (TDCPP), previously restricted from use in children’s pajamas in the 1970s due to concerns of carcinogenicity (linked to tumor formation in liver, kidney, and testes of rodents), also has been commonly used in polyurethane foam and has increasingly been used as a replacement for PBDEs [30]. These organophosphate esters have also been used as plasticizers and lubricants in various consumer products [27, 31, 32].

Little is known about exposures to these organophosphate esters. Emerging studies of exposure to TDCPP and Firemaster 550® have observed widespread concentrations in house dust samples worldwide [27, 28]; concentrations tend to vary by home and by geographic region [28, 33]. Recent biomonitoring studies have demonstrated that exposures are ubiquitous among U.S. adults [34–36]. Individual characteristics predictive of higher exposures to PFRs are not well-established, and the previous biomonitoring studies have focused primarily on pregnant women and mother-child pairs [35, 37–40]. Further elucidation of determinants of increased exposures to PFRs and the mechanisms underlying observed variability across individuals are important for potential exposure mitigation efforts and for interpreting biological monitoring data in epidemiologic studies.

PFRs have been associated with perturbations in thyroid hormone concentrations in preliminary experimental studies, with some sex-specific and compound-specific differences in the direction of the effect. Toxicological results with zebrafish and chicken embryos suggests TDCPP and TPHP have the potential to alter thyroid hormone regulation and synthesis [41–44]. Xu et al. observed a significant reduction in plasma thyroxine (T4) and triiodothyronine (T3) with TDCPP exposure in female, but not male zebrafish [45, 46]. Liu et al. reported long-term exposure to TPHP increased T3 and T4 in zebrafish [47]. A recent in vitro study observed influences on thyroid hormone transport through enhanced binding of T4 to the transport protein transthyretin in response to exposure to 6 PFRs [48]. Human studies evaluating the role of these PFR and thyroid function is quite limited with differing results. A study of U.S. men recruited from an infertility clinic observed that higher concentrations of TDCPP in house dust were linked to lower levels of T4 (*n* = 50) [49]; this association was not confirmed in an exploratory study with biological monitoring of urinary PFR metabolites in a subset of this population (*n* = 33) [50]. Preston et al. observed an association between urinary concentrations of TPHP metabolites and increased levels of T4, particularly in women (*n* = 52 U.S. men and women) [51]. Although there is a lack of direct evidence linking PFRs to thyroid cancer risk, altered thyroid hormone and thyroid stimulating hormone (TSH) levels have been associated with the risk of thyroid cancer. Epidemiologic evidence suggests that T3 and T4 exhibit tumor-promoting effects for other hormonally related cancers, such as ovarian, prostate, pancreatic cancers [52]. Epidemiologic studies have also observed associations between TSH and risk of thyroid cancer; a recent nested case-control study documented sex-specific differences in this relationship and the potential for increased risk of papillary thyroid cancer among individuals with TSH hormones outside of the normal range on either the low or high end [53, 54]. In studies of laboratory animals, TSH and thyroid hormones have also been related to tumor growth [52], and high TSH levels were linked to papillary thyroid cancer initiation in a mouse model [55]. There is an urgent need to better understand whether exposure to these chemicals leads to increased thyroid dysregulation and increased cancer risk.

The objectives of this study were to advance understanding of the human exposures and health endpoints of PFRs by (1) investigating the relationship between PFR exposure and thyroid cancer in women and (2) evaluating sociodemographic and temporal predictors of PFR exposure.

## Methods

### Study population

The current, exploratory study included a subset of 200 female, Caucasian participants from a population-based case-control study in Connecticut. The parent study has been described previously [23, 56, 57]. Cases were individuals newly diagnosed with thyroid cancer from 2010 to 2011 in Connecticut, identified through the Yale Cancer Center’s Rapid Case Ascertainment Shared Resource, the agent of Connecticut Tumor Registry. Eligibility criteria included being aged 21 to 84 years at diagnosis and having no previous diagnosis of cancer (except for non-melanoma skin cancer). Tumor subtypes were histologically confirmed (papillary, follicular, medullary, and anaplastic). A total of 462 cases participated (65.9% participation rate). Population-based controls with Connecticut addresses were recruited using a random digit dialing method. Controls were frequency-matched to cases by age (+/− 5 years); their participation rate was 61.5%. For the current analysis, 100 female papillary thyroid cancer cases were randomly selected and 100 population-based controls were matched to these cases by gender and age (5-year age groups). We focused on women due to their higher thyroid cancer incidence compared to men and on papillary thyroid tumors because they are the most prevalent. Because the parent study population was predominantly Caucasian (90%), we restricted the analysis to white women to reduce sources of heterogeneity in our analysis of 200 women. All study procedures were approved by the Human Investigations Committee at Yale (HIC # 0911005954) and the Connecticut Department of Public Health.

### Data and sample collection

After obtaining participants’ written consent, a trained interviewer administered a standardized, structured questionnaire about demographic factors, lifestyle information, medical conditions and medication use, family medical history, occupation, and diet. After the interview, spot urine samples were collected in polypropylene collection cups and stored frozen at − 80 °C in 5-ml aliquots. Interviews and urine collections were conducted from 2010 to 2013 and were scheduled at various times of day, based on participants’ availability.

### Laboratory analysis of PFR metabolites

For each participant, a 5-ml aliquot was analyzed for 6 PFR metabolites of the commonly used parent compounds tris(1,3-dichloro-isopropyl) phosphate and triphenyl phosphate: 1-hydroxy-2-propyl bis(1-chloro-2-propyl) phosphate (BCIPHIPP), bis(1-chloro-2-propyl) phosphate (BCIPP), diphenyl phosphate (DPHP), bis(1,3-dichloro-2-propyl) phosphate (BDCIPP), isopropyl-phenyl phenyl phosphate (ip-PPP), and tert-butyl phenyl phenyl phosphate (tb-PPP). Samples were analyzed using solid phase extraction (SPE) and enzyme deconjugation followed by liquid chromatography-tandem mass spectrometry, following a previously described, validated protocol [34, 35]. In brief, samples were spiked with an internal standard mixture (10 ng of d10-BDCIPP, 8.8 ng of d10-DPHP, 25 ng of d12-TCEP) and vortexed. Sodium acetate (1.75 ml of 1 M sodium acetate) was added to adjust the pH to 5. An enzyme solution was added (250 μl of 1000 units/ml μ-glucuronidase, 33 units/ml sulfatase in 0.2 M sodium acetate buffer), and samples were vortexed and incubated overnight in a 37 °C water bath. Samples were extracted and cleaned using SPE with a StrataX-AW (60 mg, 3 ml) column, and were reconstituted in 500 μl of 1:1 water:methanol. Internal standard recovery was quantified by spiking with 13C2-DPHP. Prior to analysis, the specific gravity of the samples was measured to correct for urinary dilution using a digital handheld refractometer (Atago). The extracts were analyzed using electrospray ionization (ESI) LC-MS/MS with a Phenomenex Luna C18 column on an Agilent 1100 series LC and an Agilent 6410B tandem mass spectrometer as previously described [34, 35, 37]. Laboratory researchers were blinded to case/control status.

Method detection limits (MDLs) were calculated as three times the standard deviation of the laboratory blanks, normalized to the average urine volume (3 ml). Average recoveries were 110% for dBDCIPP and 85% for dDPHP. Laboratory blind duplicates (*n* = 10) had a mean and median coefficient of variation of 19 and 15%. Specific gravity-corrected and uncorrected concentrations were highly correlated across the 6 metabolites (*r*_*Spearman*_ > 0.91) and results were very similar; therefore only SG-corrected measurements are presented, consistent with previous studies [35, 37].

### Statistical analysis

Odds ratios (ORs) and 95% confidence intervals (CIs) were calculated using logistic regression models to estimate associations between exposures to PFRs and the risk of thyroid cancer, while controlling for potential confounders. Potential confounding variables included in the final model were age (< 40, 40–49, 50–59, 60–69, ≥70), body mass index (BMI, < 25, 25–29.99, ≥30), education level, family history of thyroid cancer, previous benign thyroid disease (hyper- or hypothyroidism), and alcohol consumption (lifetime consumption of ≤12 alcoholic drinks vs. > 12 drinks). Adjustment for other variables, such as family income, smoking, and season of interview did not result in a 10% change in the ORs, and thus they were not included in the final models.

We modeled categories of exposure to individual and summed PFRs and thyroid cancer risk using tertiles, based on the distribution among controls, for the four compounds detected in > 97% of samples (DPHP, BDCIPP, ip-PPP, BCIPHIPP). We categorized BCIPP, detectable in only 46% of samples, as <MDL, ≤ median of detectable values, and > median of detectable values, using the median among controls. tb-PPP was only detected in 6% of samples and was therefore excluded from further statistical analyses. Tests for trend were based on trisected chemical concentrations in the regression models. For the compounds detectable in > 97% of samples, we also modeled the continuous concentrations of individual and summed PFRs (natural log-transformed), replacing values <MDL with one-half the detection limit. We also conducted analyses stratified by tumor size (tumor diameter ≤ 1 cm [microcarcinoma] and > 1 cm). All tests of statistical significance were two-sided with an alpha of 0.05. Analyses were conducted using R (version 3.2.4, platform x86_64-apple-darwin13.4.0).

We used multiple linear regression models to examine the relationship between natural log-transformed concentrations of PFR metabolites and self-reported individual characteristics (age, smoking status, alcohol consumption, BMI, income, education) and temporal factors (date of sample collection [continuous measure scaled to years] and season of collection [December–February, March–May, June–August, September–November]). We used step-wise backward elimination to construct models with covariates of *p*-values< 0.1, a benchmark commonly used in exposure determinants analyses.

## Results

Exposure to PFRs was ubiquitous in our study population (Table 1). ip-PPP was present at the highest concentrations, with a median concentration and interquartile range (IQR) of 2.35 ng/ml (1.33–4.51) across all samples, followed by DPHP (median = 0.82 ng/ml, IQR = 0.49–1.5), BDCIPP (median = 0.65 ng/ml, IQR = 0.31–1.6), and BCIPHIPP (median = 0.19 ng/ml, IQR = 0.09–0.45). Concentrations were similar between cases and controls (Table 1). The concentrations of the specific-gravity corrected PFRs were weakly correlated with each other (*r*_*Spearman*_ = 0.11–0.30; median = 0.21; correlations were moderate among uncorrected values (median *r*_*Spearman*_ = 0.19–0.44; median = 0.27) (Additional file 1: Table S1a and 1b).

**Table 1:** Distributions of concentrations of urinary metabolites of organophosphate flame retardants (ng/ml; specific gravity-corrected) in female papillary thyroid cancer cases and controls

Flame retardant	MDL	% Detected	Cases (*n* = 100)		Controls (*n* = 100)	
			Median	25th–75th Percentile	Median	25th–75th Percentile
BCIPP	0.185	44.5	0.242	<MDL – 0.280	<MDL	<MDL – 0.705
DPHP	0.104	97	0.802	0.457–1.549	0.843	0.506–1.450
BDCIPP	0.046	97.5	0.729	0.344–1.777	0.604	0.276–1.336
ipPPP	0.020	100	2.569	1.361–4.758	2.251	1.300–4.201
BCIPHIPP	0.001	99.5	0.160	0.084–0.377	0.214	0.093–0.564

bis(1-chloro-2-propyl)1-hydroxy-2-propyl phosphate (BCIPHIPP), bis(1-chloro-2-propyl) phosphate (BCIPP), diphenyl phosphate (DPP), bis(1,3-dichloro-2-propyl) phosphate (BDCIPP), isopropyl-phenyl phenyl phosphate (ip-PPP), and tert-butyl phenyl phenyl phosphate (tb-PPP), MDL (method detection limit)

The distributions of demographic characteristics of the female papillary thyroid cancer cases and controls are presented in Table 2. Compared with controls, cases were less educated (*p*-value = 0.009) and were more likely to have a previous benign thyroid disease (*p*-value = 0.02). Distributions of family income, and smoking status were similar among cases and controls.

**Table 2:** Distribution of selected characteristics of the papillary thyroid cancer cases and controls

	Cases (*n* = 100)	Control (*n* = 100)	
	N or %^a^	N or (%)^a^	*p*-value^b^
Age			0.47
< 40	23	17	
40–49	24	28	
50–59	39	31	
60–69	19	15	
> =70	4	9	
Years of education			0.009
High school or less	25	9	
College/Technical school	47	55	
Graduate/Professional school	25	35	
Other	3	1	
Family income			0.83
Below poverty level	6	5	
Above poverty level	71	68	
Unknown	23	27	
BMI			0.18
< 25	32	38	
25–29.99	32	38	
> =30	36	24	
Family history of thyroid cancer			0.39
No	49	49	
Thyroid Cancer	16	10	
Other Cancer	35	41	
Thyroid disease			0.02
Yes	14	4	
No	86	96	
Smoking			0.76
Yes	29	32	
No	71	68	
Alcohol consumption			0.20
Yes	37	47	
No	63	53	
Tumor diameter			–
≤ 1 cm	47	–	
> 1 cm and ≤ 2 cm	29	–	
> 2 cm	22	–	
Not available	2	–	

^a^The frequency and percentage are equivalent because the number of cases and controls each equals 100

^b^Based on chi-squared test or Fisher’s exact test when ≥1 cell has an expected frequency ≤ 5

None of the individual PFRs were positively associated with risk of papillary thyroid cancer, based on categorical and continuous forms of the exposure variables (Table 3). The odds ratios for BCIPP presented a suggestion of an inverse association with thyroid cancer risk; however all 95% confidence intervals included the null (*p*-value for trend = 0.06). Results stratified by tumor sizes were also generally null (Table 4). The odds ratios comparing the highest to lowest exposure categories for the larger tumor sizes suggested an elevated risk for DPHP, BDCIPP, IPDPP; however, confidence intervals were wide.

**Table 3:** Adjusted and unadjusted odds ratios and 95% confidence intervals for the association of urinary concentrations of organophosphate flame retardants (ng/ml; specific gravity-corrected) and papillary thyroid cancer in Connecticut women

Organophosphate flame retardant (ng/ml)	Cases	Controls	Unadjusted OR (95% CI)	p- trend	Adjusted OR (95% CI)^a^	p-trend
BCIPP						
< MDL	61	50	1		1	
≥ MDL - 0.45	26	25	0.85 (0.44, 1.66)		0.72 (0.34, 1.54)	0.06
> 0.45	13	25	0.43 (0.19, 0.91)	0.04	0.44 (0.18, 1.03)	
Continuous^b^	100	100	0.88 (0.77, 1.01)		0.89 (0.76, 1.04)	
DPHP						
< 0.59	34	33	1		1	
0.59–1.28	34	33	1.00 (0.51, 1.97)		1.02 (0.47, 2.22)	0.88
> 1.28	32	34	0.91 (0.46, 1.80)	0.80	1.06 (0.49, 2.29)	
Continuous^b^	100	100	0.94 (0.72, 1.21)		0.99 (0.74, 1.31)	
BDCIPP						
< 0.37	31	33	1		1	
0.37–0.96	28	33	1.17 (0.59, 2.35)		1.08 (0.50, 2.36)	0.46
> 0.96	41	34	1.24 (0.63, 2.46)	0.54	1.33 (0.62, 2.86)	
Continuous^b^	100	100	1.07 (0.87, 1.31)		1.07 (0.85, 1.34)	
IPDPP						
< 1.60	32	33	1		1	
1.60–3.56	26	33	0.90 (0.45, 1.83)		0.89 (0.41, 1.59)	0.66
> 3.56	42	34	1.28 (0.66, 2.51)	0.45	1.17 (0.54, 2.54)	
Continuous^b^	100	100	1.12 (0.83, 1.52)		1.06 (0.75, 1.48)	
BCIPHIPP						
< 0.12	38	33	1		1	
0.12–0.32	34	33	0.89 (0.46, 1.75)		0.92 (0.44, 1.93)	0.30
> 0.32	28	34	0.72 (0.36, 1.41)	0.34	0.66 (0.30, 1.42)	
Continuous^b^	100	100	0.85 (0.69, 1.03)		0.82 (0.65, 1.01)	
Summed PFR						
< 4.10	35	34	1		1	
4.10–7.95	29	33	0.85 (0.43, 1.70)		0.94 (0.43, 2.00)	
> 7.95	36	33	1.06 (0.54, 2.07)	0.87	1.07 (0.50, 2.31)	0.86
Continuous^b^	100	100	0.96 (0.71, 1.31)		0.93 (0.65, 1.33)	

bis(1-chloro-2-propyl) 1-hydroxy-2-propyl phosphate (BCIPHIPP), bis(1-chloro-2-propyl) phosphate (BCIPP), diphenyl phosphate (DPP), bis(1,3-dichloro-2-propyl) phosphate (BDCIPP), isopropyl-phenyl phenyl phosphate (IP-PPP), and tert-butyl phenyl phenyl phosphate (tb-PPP), odds ratio (OR), 95% confidence interval (95% CI), PFR (organophosphate flame retardant)

^a^Adjusted for age, BMI, education level, family history of thyroid cancer, previous benign thyroid disease, and alcohol consumption

^b^Odds ratio calculated for each 1og of 1 ng/ml

**Table 4:** Odds ratios and 95% confidence intervals for the association of urinary concentrations of organophosphate flame retardants (ng/ml; specific gravity-corrected) and microcarcinomas and larger tumor papillary thyroid cancer in Connecticut women

	Microcarcinomas (Tumor Diameter ≤ 1 cm)					Larger Tumor Size (Tumor Diameter > 1 cm)				
Organophosphate Flame Retardant (ng/ml)	Cases	Controls	OR^a^	95% CI	p- trend	Cases	Controls	OR^a^	95% CI	p- trend
BCIPP										
< MDL	32	50	1			28	50	1		
≥ MDL - 0.45	9	25	0.52	(0.18, 1.38)	0.06	16	25	0.99	(0.38, 2.56)	0.31
> 0.45	6	25	0.38	(0.12, 1.09)		7	25	0.51	(0.15, 1.56)	
Continuous^b^	47	100	0.84	(0.69, 1.03)		51	100	0.94	(0.77, 1.14)	
DPHP										
< 0.59	17	33	1			16	33	1		
0.59–1.28	15	33	0.87	(0.33, 2.29)	0.61	18	33	0.94	(0.33, 2.67)	0.57
> 1.28	15	34	0.78	(0.29, 2.05)		17	34	1.32	(0.49, 3.64)	
Continuous^b^	47	100	0.86	(0.56, 1.26)		51	100	1.08	(0.76, 1.55)	
BDCIPP										
< 0.37	15	33	1			14	33	1		
0.37–0.96	15	33	0.76	(0.28, 2.03)	0.90	18	33	1.02	(0.36, 2.86)	0.49
> 0.85	17	34	1.03	(0.41, 2.65)		19	34	1.43	(0.51, 4.08)	
Continuous^b^	47	100	0.98	(0.74, 1.31)		51	100	1.12	(0.84, 1.51)	
IPDPP										
< 1.60	17	33	1			13	33	1		
1.60–3.56	12	33	0.79	(0.30, 2.08)	0.99	16	33	1.00	(0.35, 2.84)	0.52
> 3.56	18	34	1.01	(0.39, 2.55)		22	34	1.37	(0.50, 3.83)	
Continuous^b^	47	100	1.06	(0.70, 1.60)		51	100	0.98	(0.62, 1.54)	
BCIPHIPP										
< 0.12	18	33	1			20	33	1		
0.12–0.32	18	33	1.06	(0.44, 2.57)	0.25	15	33	0.81	(031, 2.10)	0.27
> 0.32	11	34	0.54	(0.19, 1.45)		16	34	0.57	(0.21, 1.52)	
Continuous^b^	47	100	0.79	(0.57, 1.06)		51	100	0.77	(0.59, 1.00)	
Summed PFR										
< 4.10	20	34	1			14	34	1		
4.10–7.95	12	33	0.65	(0.25, 1.66)		17	33	1.37	(0.50, 3.83)	0.55
> 7.95	15	33	0.75	(0.29, 1.93)	0.53	20	33	1.37	(0.50, 3.79)	
Continuous^b^	47	100	0.77	(0.47, 1.22)		51	100	1.02	(0.66, 1.57)	

bis(1-chloro-2-propyl) 1-hydroxy-2-propyl phosphate (BCIPHIPP), bis(1-chloro-2-propyl) phosphate (BCPP), diphenyl phosphate (DPP), bis(1,3-dichloro-2-propyl) phosphate (BDCPP), isopropyl-phenyl phenyl phosphate (IP-DPP), and tert-butyl phenyl phenyl phosphate (tb-PPP), odds ratio (OR), 95% confidence interval (95%CI), PFR (organophosphate flame retardant)

^a^Adjusted for age, BMI, education level, family history of thyroid cancer, previous benign thyroid disease, and alcohol consumption

^b^Based on log-transformed PFR concentrations; odds ratio calculated for each 1og of 1 ng/ml

Our exposure determinants analysis demonstrated a relationship between higher BMI and two of the urinary PFR metabolite concentrations (BDCIPP, ip-IPP) (Table 5). Compared to women in the normal BMI categories, women in the obese BMI categories had approximately 1.7-times higher levels of all the PFRs and summed PRF. We observed lower concentrations of BCIPP and the summed PFRs in 2011–2013 compared to 2010. We observed some seasonal differences with BDCPP, ip-IPP and the summed PFR concentrations highest in summer and lowest in winter. Ever smokers had lower concentrations of BDCIPP and ever alcohol consumers had higher concentrations of DPHP. No associations with age, income, or education were observed.

**Table 5:** Multiplicative change in organophosphate concentration (*e*^β^) (specific-gravity corrected) with respect to potential exposure predictors

Potential Predictors^a^	BCIPP	DPHP	BDCIPP	IPIPP	BCIPHIPPP	Sum
BMI (ref: Normal)						
Overweight	–	–	0.98 (0.62, 1.5)	1.4 (1.0, 1.9)	–	1.2 (0.87, 1.6)
Obese	–	–	1.6 (1.0, 2.6)	1.7 (1.3, 2.4)	–	1.7 (1.2, 2.3)
Year (Ref: 2010)						
2011	0.32 (0.16, 0.64)	–	–	–	–	0.68 (0.49, 0.93)
2012	0.27 (0.12, 0.61)	–	–	–	–	0.64 (0.43, 0.94)
2013	0.57 (0.23, 1.5)	–	–	–	–	0.64 (0.42, 0.98)
Season (Ref: Winter)						
Spring	–	–	1.4 (0.80, 2.5)	1.5 (1.0, 1.9)	–	1.4 (0.99, 2.1)
Summer	–	–	2.4 (1.3, 4.3)	1.7 (1.1, 2.5)	–	1.7 (1.1, 2.4)
Fall	–	–	1.2 (0.69, 2.2)	1.6 (1.0, 2.3)	–	1.2 (0.83, 1.8)
Ever Alcohol (Ref: Never)	–	1.4 (1.0, 1.9)	–	–	–	–
Ever Smoker (Ref: never)	–	–	0.59 (0.40, 0.89)	–	–	–

^a^The following variables were not associated with any metabolite concentrations: education, below vs. above poverty level, or age at the level of *p* < 0.1

## Discussion

To our knowledge, this is the first study evaluating biomarkers of exposure to PFRs and risk of thyroid cancer. In our analysis of adult women, we found no association between exposure to PFRs and risk of papillary thyroid cancer. We observed that urinary PFR levels vary by season and BMI.

In general, the concentrations of PFR metabolites in our adult, female Connecticut population (2010–2013) were lower than those observed in adults (mostly pregnant women) in other time frames and geographic areas, as reviewed in a recent study [58]. The exception was ip-PPP; our observed concentrations were similar or higher to the few recent cohorts measuring that specific compound. The lower observed concentrations in urinary PFR metabolites in our study could be attributable to differences in toxicokinetics (e.g., kidney function) or exposure patterns among our population of women, who were not pregnant and had median age 10–20 years greater than the previous studies of pregnant women. Differences could also reflect geographic variations in flammability standards.

Exposure to PFRs was widespread in our population. Our exposure determinants analysis revealed associations between higher PFR concentrations and increased BMI. Possible explanations include the obesogenic potential of PFRs themselves [59] or common activities or behaviors leading to both higher BMI and higher exposure [58]. Neither income nor education were linked to urinary PFR concentrations; however, the participants were generally white, well-educated women living above the poverty line, reducing our power to fully examine these demographic patterns. We also observed a strong seasonal pattern, with higher urinary PFR levels in the summer, consistent with a previous exposure determinants analysis [58]. This relationship could reflect increased volatilization of PFRs and subsequent increased inhalation exposure in warmer months [60], increased dermal exposure due to greater surface area of exposed skin and increased perspiration, or possibly incomplete adjustment for relative dehydration in summer months (i.e., lower dilution of samples), although all samples were corrected for specific gravity.

Our study has several strengths, including its novel study question, population-based study design, and rapid and thorough identification of incident cases using the Connecticut Tumor Registry. All the cases were histologically confirmed to minimize misclassification of outcomes; information on tumor size was available for analysis. Detailed information on potential confounding factors were collected and controlled for in the analysis. Finally, the use of a biological marker of PFR exposure is an objective exposure measure. Finally, our study provides additional exposure data on these ubiquitous, endocrine-disrupting chemicals.

Several limitations warrant consideration. The most important is that our samples were collected post-diagnosis. As with all retrospective case-control studies of biomarkers and chronic disease, the exposures may not be representative of past exposure during etiologically relevant time windows, which have not yet been established for thyroid cancer. Additionally, measurements of PFRs in spot urine collections may not be representative of usual, long-term exposure due to their short half-lives. Intra-class correlation coefficients for repeated urinary BDCIPP and DPHP concentrations measured over 3 to 9 months range from 0.3 to 0.7 [36, 61]. The resulting misclassification from post-diagnosis, spot urine samples would likely attenuate risk estimates. Unfortunately, no long-term biomarkers of exposure exist. Another potential limitation with a post-diagnosis urine sample is that disease status or chemo- or radio-therapy could affect the concentrations of PFRs, though we do not expect this. Unlike other cancer sites, papillary thyroid cancer patients are treated either by surgery alone or surgery and postoperative ^131^I treatment. This is an extremely effective and specific treatment for thyroid cancer; no other organs or cells are affected by the radioactive iodine. As such, the treatment for papillary thyroid cancer patients is less likely to affect the urinary levels of PFRs. A nested case-control or cohort study with repeated samples would be needed to overcome these limitations. Measurement of PFRs in residential dust samples could also be used to as a proxy for past exposures, as they may be representative of longer time periods. However, these measures only capture the home environment and do not capture exposures occurring in vehicles, the workplace, or via dietary intake.

Limitations related to our population were our moderate number of cases (*n* = 100). Also, though our study was population-based, the generalizability is limited to those populations with similar exposures and to Caucasian women. Future studies could explore whether there are associations between PFR exposure and risk of thyroid cancer in men, women of other races and ethnicities, and populations in other geographic regions.

## Conclusions

The widespread PFR exposures within our population were linked to BMI and season. Despite the evidence of disruption of thyroid homeostasis by PFRs, this study does not provide support for an increased risk of thyroid cancer associated with exposure to PFRs, measured at the time of diagnosis. Given the biologic plausibility, the relationships between PFR exposure and thyroid cancer risk warrant further investigation in a larger study population with additional biomarker measurements.

## Additional file

Additional file 1: **Table S1a.** Spearman correlations among organophosphate flame retardants (specific gravity-corrected) (*n* = 200). **Table S1b.** Spearman correlations among organophosphate flame retardants (not specific gravity-corrected) (*n* = 200). (DOCX 17 kb)

## Abbreviations

95% CI: 95% confidence intervals
BCIPHIPP: Bis(1-chloro-2-propyl) 1-hydroxy-2-propyl phosphate
BCIPP: Bis(1-chloro-2-propyl) phosphate
BDCIPP: Bis(1,3-dichloro-2-propyl) phosphate
BMI: Body mass index
DPHP: Diphenyl phosphate
ip-PPP: Isopropyl-phenyl phenyl phosphate
OR: Odds ratio
PBDE: Polybrominated diphenyl ether
PFRs: Organophosphate flame retardants
SPE: Solid phase extraction
T3: Triiodothyronine
T4: Thyroxine
tb-PPP: Tert-butyl phenyl phenyl phosphate
TDCPP: Tris (1,3-dichloro-isopropyl) phosphate
TPHP: Triphenyl phosphate
U.S.: United States
