# Supplementary material for: A case-control study of exposure to organophosphate flame retardants and risk of thyroid cancer in women
Source: BMC Cancer. 2018 Jun 5;18:637. doi: 10.1186/s12885-018-4553-9 (PMC5989427; doi:10.1186/s12885-018-4553-9)
Supplement: Supplementary file 1 — Table S1a. Spearman correlations among organophosphate flame retardants (specific gravity-corrected) (n = 200). Table S1b. Spearman correlations among organophosphate flame retardants (not specific gravity-corrected) (n = 200). (DOCX 17 kb) [file 12885_2018_4553_MOESM1_ESM.docx]

| Supplementary Table 1a. Spearman correlations among organophosphate flame retardants (specific gravity-corrected) (n=200). | | | | |
| --- | --- | --- | --- | --- |
|  | BCIPP | DPHP | BDCIPP | IPDPP |
| DPHP | 0.11* |  |  |  |
| BDCIPP | 0.20** | 0.30*** |  |  |
| IPDPP | 0.21** | 0.22** | 0.22** |  |
| BCIPHIPPP | 0.25*** | 0.18* | 0.21** | 0.17* |

*p<0.05, **p<0.01, ***p<0.001

| Supplementary Table S1b. Spearman correlations among organophosphate flame retardants (not specific gravity-corrected) (n=200). | | | | |
| --- | --- | --- | --- | --- |
|  | BCIPP | DPHP | BDCIPP | IPDPP |
| DPHP | 0.19** |  |  |  |
| BDCIPP | 0.27*** | 0.44*** |  |  |
| IPDPP | 0.33*** | 0.44*** | 0.38*** |  |
| BCIPHIPPP | 0.27*** | 0.25*** | 0.26*** | 0.26*** |

*p<0.05, **p<0.01, ***p<0.001
